# Longing for ground in a ground(less) world: a qualitative inquiry of existential suffering

**DOI:** 10.1186/1472-6955-10-2

**Published:** 2011-01-27

**Authors:** Anne Bruce, Rita Schreiber, Olga Petrovskaya, Patricia Boston

**Affiliations:** 1 School of Nursing, University of Victoria, Victoria, British Columbia, Canada; 2 Director, Division of Palliative Care, Department of Family Practice, University of British Columbia, Vancouver, British Columbia

## Abstract

**Background:**

Existential and spiritual concerns are fundamental issues in palliative care and patients frequently articulate these concerns. The purpose of this study was to understand the process of engaging with existential suffering at the end of life.

**Methods:**

A grounded theory approach was used to explore processes in the context of situated interaction and to explore the process of existential suffering. We began with in vivo codes of participants' words, and clustered these codes at increasingly higher levels of abstractions until we were able to theorize.

**Results:**

Findings suggest the process of existential suffering begins with an experience of groundlessness that results in an overarching process of *Longing for Ground in a Ground(less) World*, a wish to minimize the uncomfortable or anxiety-provoking instability of groundlessness. Longing for ground is enacted in three overlapping ways: by turning toward one's discomfort and learning to let go (*engaging groundlessness*), turning away from the discomfort, attempting to keep it out of consciousness by clinging to familiar thoughts and ideas (*taking refuge in the habitual*), and learning to live within the flux of instability and unknowing (*living in-between*).

**Conclusions:**

Existential concerns are inherent in being human. This has implications for clinicians when considering how patients and colleagues may experience existential concerns in varying degrees, in their own fashion, either consciously or unconsciously. Findings emphasize a fluid and dynamic understanding of existential suffering and compel health providers to acknowledge the complexity of fear and anxiety while allowing space for the uniquely fluid nature of these processes for each person. Findings also have implications for health providers who may gravitate towards the transformational possibilities of encounters with mortality without inviting space for less optimistic possibilities of resistance, anger, and despondency that may concurrently arise.

## Background

Existential and spiritual concerns are fundamental issues in palliative care and patients frequently articulate these concerns. Although research on existential concerns has slowly emerged in recent years, there remains a scarcity of studies about how existential issues are understood, managed and treated in palliative care settings [1]. As the metaphoric landscape of palliative care shifts and the field matures within a broader context of technological and scientific advances aimed at prolonging and enhancing quality of life [2], palliative care is increasingly concentrated on medicalization [3]. This focus presents the complex issue of existential suffering as a unique challenge to the palliative care community that is only just beginning to understand existential suffering as a uniquely subjective response [4].

Existential distress or suffering has been described as a condition where morbid suffering in patients may include concerns related to hopelessness, futility, meaninglessness, disappointment, remorse, death anxiety, and a disruption of personal identity [5]. Arthur Frank [6] has stated "suffering is the unspeakable, as opposed to what can be spoken; it is what remains concealed ... beyond what is tangible even hurtful" (p. 355). Although there have been multiple attempts to define and understand existential suffering, this debilitating symptom in the palliative care context remains a widely discussed yet ill defined concept [7,8]. Moreover, existential suffering often remains a neglected symptom of overall suffering [9,10]. And although researchers have proposed that qualitative research is the methodology of choice to understand subjective experience relating to meanings, patterns and relationships [11], few such qualitative studies exists related to the end of life. According to Henoch and Danielson [1], studies to date on existential suffering largely include randomized control trials, case studies, pre and post test quantitative designs, and descriptive studies. For instance, Wilson et al.'s study [12] employed a combination of a comparative-correlational design and a content analysis of semistructured interviews to examine suffering in patients with advanced cancer.

There is a scarcity of qualitative research on the inner life domains of spirituality and existential concerns in actual palliative care settings [13]. More specifically, there is little research evidence around the processes by which existential suffering is understood and managed in palliative care. One recent example includes a grounded theory study that explored existential distress in patients with advanced cancer vis-a-vis notions of hope and meaning from a perspective of palliative care professionals working in a Christian hospital in Japan [14]. In contrast, the purpose of this study, situated in Canada, was to understand the process of engaging with existential suffering at the end of life from the perspectives of health care staff, patients, and family care providers.

## Methods

We used grounded theory, a qualitative, systematic approach used to explore processes in the context of situated interaction, to explore the process of existential suffering. It involves the concurrent collection and analysis of data to formulate theories that are grounded in the worlds of the participants [15,16]. The intent of grounded theory is to develop a theory that explains the situated actions and interactions of participants as they experience, engage with, and manage, the phenomenon of study. In reporting these findings, we are "grounded-theorizing" [17] rather than presenting a theory that might be viewed as static. This is in keeping with one of the basic precepts of the method, that grounded theories are modifiable in the event of new data coming to light.

### Participants

We used purposive sampling and snowballing to obtain a sample of 22 participants experienced and knowledgeable with end of life issues. Participants who identified themselves as having experience with existential suffering at the end of life were included in the study. Participants included 6 people with a cancer illness, 6 family caregivers and 10 health care professionals (nurses, chaplains, social workers, physicians). To explicate the phenomenon of study fully and in keeping with grounded theory precepts [15,16], we sought participants with a wide range of experience of existential suffering. The varied perspectives on the existential suffering enabled us to "flesh out" its dimensions and properties, and thus, the theory articulated here represents the *phenomenon of existential suffering *rather than that of the experiences of the different groups of participants.

### Data Collection

We conducted a series of semi-structured interviews lasting between 60-240 minutes. Wherever possible, we spoke with people in person; in addition we conducted 3 telephone interviews with people living too far away to travel. Three categories of information about participants' experiences were sought. These included: 1) the nature of existential suffering; 2) responses that arise as a result of existential suffering; and 3) perceptions of what exacerbates or reduces existential suffering. The interviews began with an open-ended question including, "Tell me what it has been like since receiving your diagnosis?" or with care providers, "Tell me what it is like being with patients who experience intolerable non-physical suffering?" This was followed by prompts such as "can you tell me more about that?" The purpose of this approach was to elicit the person's perspective with as few prompts as possible. All interviews were recorded and transcribed verbatim by a transcriptionist. We conducted two follow up interviews (for a total of 24 interviews) and have engaged in extensive email discussions with two family caregiver participants.

### Data Analysis

In grounded theory, data analysis and data collection occur iteratively, and therefore data analysis began with the first interview and continued throughout the study. Repeatedly we listened to interviews and read transcripts, individually and collectively coding at multiple levels of abstraction. We began with *in vivo *codes of participants' words, and clustered these codes at increasingly higher levels of abstractions until we were able to begin theorizing. In team meetings we discussed the data and kept notes for future reference. Throughout the process we wrote memos to clarify concepts and hypothesize connections between ideas, in keeping with grounded theory traditions.

### Trustworthiness

To ensure trustworthiness of the study and its findings, verbatim transcription, constant comparison, and persistent and prolonged engagement with the data were used [18]. In addition, we used peer debriefing within a grounded theory methodology seminar and solicited feedback from health professionals at palliative care conferences. The use of transcribed data can be associated with potential bias [19], and to compensate, we listened to the tapes repeatedly, while reading and re-reading the text. A grounded theory is said to be sound when it has "fit, work, and grab" [15]. That is, the theory fits the data and works to explain the variation within the data set. The notion of "grab" is used to describe the situation in which the findings are immediately recognizable to those who are knowledgeable about the phenomenon of study, in this case, existential suffering at the end of life.

### Ethics

The study was conducted in accordance with the Canadian Tri-Council (1998) guidelines for research involving humans, including informed consent. Because of the sensitive nature of interviews, we drew on our professional communication skills as nurses (RS, OP) with palliative care clinical experience (AB, PB) to ensure the emotional comfort of the participants. Before the study was underway, approval of the Human Research Ethics Board of the University of Victoria was received.

## Results

We did not begin with a definition of existential suffering, but instead, sought participants' understanding of what it meant for them. It became clear that participants' understandings of existential suffering were as varied as we see in the literature [1,20,21]. Many felt that our very existence as human beings necessarily involves suffering, "to be fully human means to suffer." Over the course of a lifetime, we experience "little deaths": significant losses that precipitate suffering. However, when faced with one's own death, suffering takes on another dimension. It may be that language is inadequate to talk about acute moments, or "raw experiences" of existential suffering because these experiences represent a gap, a space within the continuity of life as we normally live it. One participant spoke of how the language of psychology or social science is inadequate, and he turned to poetry, literature, and the metaphoric language of religious texts to talk about suffering.

### Groundlessness: The problem

An essential task of the grounded theorist is to identify the often-unarticulated basic social problem or challenge shared by participants. For these participants, the challenge was experiencing *Groundlessness *that results from what one person called "being shaken to the core". Patients and family members experienced being shaken to the core on learning news of a terminal diagnosis. Balfour Mount's [22] description of the existential moment could apply equally to the groundlessness that comes with being shaken to the core: "A crack appears in our carefully crafted concept of reality... The very nature of reality is experienced in a new way. We are sucked into the startling realization that the rules of the game are not what we had imagined" (p. 93-94).

Groundlessness is a time and place of raw experience and frayed emotions. Participants used emotional terms in describing it, talking about fears, losses, questioning, worrying, discontinuity, pain, despair, frustration and anger. They also used "un" terms such as feeling undone, unravelled, or unhinged to describe being groundless. Participants spoke of recognizing life is ending, having a profound sense of hopelessness, being unable to reconcile their experience with their spiritual faith, not understanding why God is doing this, having ones' belief system shattered, experiencing extreme dissonance. Therese, a physician, provides an example of anguish and the types of questions a middle-aged patient experiencing groundlessness asked: "Why me... why now when I finally have my life together... why now when I've worked so hard to be a well person--where's the justice in this? Where's the fairness? Is this happening just because my life's always been unfair? I finally thought I had it figured out and then this..."

Others conveyed groundlessness through their feelings of deep despair and an unmalleable grief. One participant described it as "the sense of hopelessness that is quite unlike anything one has experienced before. Past coping mechanisms to make sense no longer work".

Caregivers also experienced groundlessness. In situations when the patient's suffering seemed irresolvable and no peaceful end was possible, an infectious or rippling suffering was evoked for some professional and family caregivers. This groundlessness was characterized as resonating suffering, as one caregiver shared: "[the] struggle in someone else's life opens up fears and anxieties about the transient nature of our own lives here on earth... Maybe not just the fact that we will die, but the fact that we may suffer or face fear and pain".

As illustrated, caregiver suffering was heightened as the patient's suffering endured despite all efforts to relieve it. When deprived of the ground of familiar meanings and connections, patients, families, and professional caregivers all engage, albeit somewhat differently, in the search for stability and grounding.

### Longing for Ground in a Ground(less) World: The Process

No matter the words or metaphors used, experiencing groundlessness is profoundly distressing, in that a patient's world is shattering and his/her fundamental beliefs are called into question. Experiencing groundlessness involves suffering, what one participant called "suffering our spirits", and leads to the search for peace or stability, which we have named *Longing for Ground in a Ground(less) World*. This is the basic social process, a type of core category [16,17], by which participants make sense of and ameliorate their groundlessness. We have put "less" in parentheses to designate that the perception of being grounded or groundless is fluid and constantly shifting. Moreover, without parentheses, this phrase would sound too futile and deterministic; broken down into two parts, ground-less embraces possibilities for multiple interpretations. The basic social process of *Longing for Ground in a Ground(less) World *is comprised of three categories: engaging groundlessness, taking refuge in the habitual, and living in-between. The process involves moving between *engaging groundlessness*, in which people turn toward the discomfort of groundlessness and learn to let go; *taking refuge in the habitual*, in which people turn away from the discomfort, attempting to keep it out of consciousness by clinging to the familiar; and *living in-between*, in which people may create a balance within groundlessness and potentially find comfort in the instability.

#### Engaging groundlessness

Engaging groundlessness is moving into the discomfort of being groundless and working with that instability. It may be that life has prepared people by giving them "little deaths"--losses that have happened along the way, so that the end of life, though big, is in some sense, "just another death". Engaging groundlessness is based on a belief that groundlessness is workable, that one can learn to let go. This involves learning how to work with and make sense of what life presents now, so that what was normal before the diagnosis no longer applies. Instead, participants continuously renegotiate and reconfigure what is normal, as well as the sense of self, of relationships, and so forth. For example, participants spoke of learning to let go and live with ambiguity. They spoke of (re)connecting or (re)normalizing as ways of making new meanings of what is happening, of living in the flux, which is in some sense waking up to the uncertainty of human existence that has been there all along.

We heard many stories from health care professionals about working with patients experiencing existential suffering, helping them find or create new meanings as they narrated their lives. One chaplain, for example, spoke of "finding the key" to unlock patients' suffering and anger that distanced others and helping patients reconnect with their previous lives. Another chaplain described how she searched for the metaphors used by patients, for example, "looking beyond the gate" or "playing the hand one is dealt", and using such language to open up discussion with patients seemingly locked in their suffering.

On the other hand, the belief that it is the caregiver's responsibility to offer "some sort of reassurance, something [the dying] can grab onto" to help relieve patients' suffering is not necessarily helpful, and Sara, a family caregiver, quickly grew tired of people who just "want to make nice" and avoid the difficult reality of the situation. For Sara, as for others, it was important to face the reality of death, including one's own death.

Yet the process of engaging groundlessness is not constant. For example, Daniel, who has a terminal diagnosis, spoke about needing times when he disengages: "I don't know if your mind shuts down and you don't want to believe it, or I mean right now I don't feel like anything's going on. Like, I'm not sick, I don't have anything, so it's not tangible where you can put your hands on it. So, it's like, not there. It's mind-boggling. It's really hard to grasp sometimes." In this way, engaging groundlessness can involve stepping away from the flux at times when it becomes "too much".

Similar to Daniel, a social worker described the recurring critical moments that make up the process of engaging groundlessness as experienced by providers: "I think that as professionals we're making a choice almost in every encounter: are we going to be open, to being touched and then hurting? And feeling pain and loss ourselves? Or are we not? And we don't necessarily make that decision once and then keep the doors closed or the doors open--our own emotional doors--from that moment on, forever and ever. We open them and close them as our own sense of vulnerability increases or decreases." The metaphors of the mind shutting down and the closed doors tellingly show that, even though letting go and living in groundlessness and ambiguity may have become the new reality for both patients and care providers, this engagement with this new reality is untenable for very long.

Engaging groundlessness requires effort and moment by moment decisions about whether, how, and how much to engage at any given point in time. As seen in the quotes above, engaging is a process rather than a continuous state of being, because it seems impossible to engage fully on a constant basis. And, although it might seem as if engaging groundlessness would relieve existential suffering, the process of suffering and the groundlessness of one's world continue as losses accumulate and one's ability to actively engage groundlessness diminishes.

#### Taking refuge in the habitual

Taking refuge in the habitual is in some sense the opposite of engaging groundlessness, as it is turning away from the instability of groundlessness and seeking security in the familiar. Taking refuge in the habitual involves skirting, or trying to avoid the existential questions, those "questions some of us refuse to ask", that inevitably arise when facing a terminal illness. In the face of questions that challenge us to examine the very core of our beings and the meanings of our lives, it can be easier to find comfort in our usual patterns and ways of thinking. Patients, families, and care providers all spoke of the need to retreat from the inevitable and take relief, however temporary, in the known.

Taking refuge in the habitual is a way of dealing with suffering by connecting to familiar ideas or conceptual models of how the world is/should be, and who one is within it. Taking refuge in the habitual is a way of *Longing for Ground *within the emotional maelstrom of existential suffering by using cognitive means, seeing the world through familiar eyes and relating to it as if nothing has changed. For patient and family caregiver participants, taking refuge in the habitual involves relating to life as it was known before the diagnosis, and playing by the recognizable rules of the pre-existing narrative structure of how the world works. These familiar ideas are challenged by the diagnosis, and yet it is possible to hold on, sometimes desperately, so that we use our ideas to surround and protect our core sense of self. For example, a respected professional, after being diagnosed with a terminal illness, accepted a new, prestigious position, relocated to a distant city, and subsequently died shortly after. For this person, clinging to a professional status was clearly important. Taking refuge in the habitual is often about control, distancing, and disconnection, and ultimately may prove illusory.

Sometimes people engage the world through strongly held beliefs that provide solace, but that may no longer work in the current reality. For example, Daisy, a social worker, described what can happen when at the end of life people realize that their previously unassailable religious beliefs do not hold them: "Some of the *most *profound despair that I've witnessed has been with people who have had strong spiritual faith, and with this [terminal illness] happening to them they cannot reconcile the two. They can't understand why God is doing this, if their belief [is] that God is an interventionist God, that God answers prayers--they can't understand that." Daisy also described a situation in which a man who had experienced a "born-again" event could not understand how the "God that he so strongly believed had reached out and saved him [before], could now allow him to die and leave his little children without him".

Patients spoke of turning away from groundlessness by engaging themselves elsewhere. For example, one participant with cancer described being disconnected from himself and what was happening around him by escaping into what he called "mindgames". At the same time, he wondered why he was doing this, and recognized that he was engaging with a difficult situation in his habitual fashion, and disconnected from the situation by using thinking as a way of controlling his fears by taking himself out of the picture.

Taking refuge in the habitual is difficult in the face of the inevitably compounding losses at the end of life that make it harder to relate to the world through a veil of ideas that can no longer obscure those losses. Yet, as a way of *Longing for Ground*, taking refuge in the habitual can endure even when it no longer seems to work to make sense of what is going on. As a refuge, it holds the promise of relief of suffering; however, suffering intensifies as one realizes the impossibility of staying in this refuge forever. The realization begins to dawn that the "old" solid ground is an illusion and one is propelled to search for new ground in the form of new hope, new meaning, or another untested illusion. One participant described the commonality of the human condition and the futility of trying to control, circumscribe, and contain the groundlessness of dying in this way: "I think a lot of times what people struggle with are questions and not answers and, well, if they struggle with it, why shouldn't we? So you're left with a question... You're left with a 'well, I don't know.' Well there's a lot of 'I don't knowness' about life. Why should you be spared that? Why should everything be all neatly wrapped up in a box, you know?"

In the end, taking refuge in the habitual is a way of avoiding the inevitability of death and the suffering it entails, albeit temporarily. The ideas and the solid ground they seemed to provide prove illusory as the urgency of one's death overtakes all. In many ways, the sense of being on solid ground provided by taking refuge in the habitual only masked the reality that was there all along: we will all inevitably die, and that dying will entail suffering our spirits.

Grey, a social worker, described the incompatibility between our notion of being in control and the realities of death: "Caregivers deem it their responsibility to accentuate or augment [patients'] experience of control. What's the assumption? That you're maintaining a person's sense of control over their lives. What's the assumption of that? That they've enjoyed that in the pre-morbid state. What I'm trying to suggest is, that's an illusion that is so old - I mean it [control] is entrenched but it doesn't mean it's not an illusion. You are never in control."

Taking refuge in the habitual is a way of dealing with existential suffering that involves reliance on one's usual patterns and familiar grounds. Participants indicated that everyone takes refuge in familiar ideas at least occasionally when experiencing the groundlessness of existential suffering, some more than others. By taking refuge in the habitual, we are enabled to engage with our worlds as though nothing has happened (and yet it has), and set aside (for a time) the inevitability and horror of facing our own immanent mortality.

#### Living in-between

Living in-between represents the place where suffering at the end of life is reconsidered as a person actively navigates the shifting passage between living and dying. Living in-between, people negotiate the ambiguities of both engaging groundlessness with its letting-go, and seeking refuge in the habitual while holding-on. In other words, living in-between is an attempt to become comfortable with constant shifting within the experiences of losing ground, letting go of that loss, finding a new frame of reference only to realize that it, too, is a temporary ground that will slip away.

Participants' difficulty with talking about *living in-between*, and our difficulty with supporting our theorizing with quotes, may be explained by the very nature of this process that we seek to describe. We seek to dress in words that which might lie beyond language: a place where people attempt to make sense of new realities, and the painful shifts from losing ground to an illusory idea of feeling that ground again.

Nevertheless, living in-between is a way of living in the flux of knowing that in many ways things are profoundly changed, yet at the same time they are not. Living in-between, one might think: "I am a different person (cancer patient), and yet...it's still me. *I *haven't changed--or have I?" The circumstances have changed, the dreams and plans have changed, the priorities have changed, and yet it is still this life where we are the same.

Daniel, who was recently diagnosed with terminal cancer, seems to dwell in-between his "normal" and "changed" states: "I mean as much as a life-threatening illness changes you, it doesn't. Changes maybe the thoughts and certain things you do, but you as a person, I try to continue on as normal as possible, just to keep that normalcy. So I don't wake up in the morning and go, 'Oh well, I should do this today 'cause it could be my last day'". Life comes to an abrupt halt and yet we carry on as if it is normal. We behave as if there is an objective reality because it is too much otherwise. Therefore we need to carry on as normal, knowing that it is not, and yet it cannot be otherwise. What else can you do?

At the same time that the immediacy of one's own death may be filled with dread, some participants recognized opportunities for joyful experiences. There is a recognition that living in the knowledge of death enriches life; the experience itself could bring richness in family relationships previously unknown. In more than one family, the diagnosis of a terminal cancer opened opportunities for renewed, stronger relationships. In this way, the shock of facing death can bring gifts.

Although existential suffering can lead to openings and insights and, in Patricia's view, through suffering we become more human--this is not always the case. Raw suffering is more difficult to articulate and it is expressed on many levels. Leah, a nurse, shared her experience of working with a terminally-ill woman tormented by the realization that she had not loved enough in her life. The woman was inconsolable and in "dire pain that a pain pill would not take away": "...She would lament incredibly and wake up sobbing and crying. What ended up happening was the nurses would spend time holding her and touching her and caressing her and soothing her and...just sort of offering what we could in that moment until she died... All we could demonstrate is loving and compassion to her in the moment and hopefully that made a difference. Whether you ever really do or not, you're not sure... It was her spirit suffering..."

In addition to suggesting that suffering at the end of life does not necessarily become a positive transformational experience, the above quote reveals health care providers' feeling of ambiguity and in-between-ness. Resonating suffering of care providers is often an in-between place of knowing they have done all they could, yet not knowing or feeling if that was enough. Health care providers whom we interviewed spoke of learning to "be okay with not being okay." A social worker shared her wisdom gained through many moments of resonating suffering that it is okay to feel inadequate when faced with existential questioning of dying persons. A sense of caregivers' vulnerability and inadequacy brought about by patients' existential despair may, in fact, be the inherently human experience of witnessing death. What is more intriguing and paradoxical, this sense of a profound vulnerability evoked in caregivers, far from presenting an impediment to (re)connecting with a dying person, provides an opening for meaningful and authentic connection. Resonating suffering can be the only common experience between the caregiver and the dying patient.

There may be yet another sense of in-between-ness. From the interviews we glimpsed that suffering that permeates the struggles to make new meaning and remain in control over one's life, and the relaxation into letting go--all that suffering sometimes ceases to exist for the dying person. Perhaps a person finds solace in knowing that his or her life was meaningful and well-lived; perhaps the meaning is re-defined or no longer important. But perhaps the whole human frame of reference is transformed, and the notions of life or the world as being meaning*ful *or meaning*less *become empty. Searching for meaning is like longing for ground in the world that is groundless.

For example, Patricia described a patient's family members who were trying in vain to make sense of, to find a meaning in, the dying woman's unexplainable lingering between life and death. In hindsight, Patricia reflected that sometimes life and the world just *are *what they *are*, and existential suffering at end of life is just that--connected to the finitude of human existence and to letting go of the attachments that were formed throughout life.

## Discussion

Findings suggest the process of existential suffering begins with an experience of groundlessness, when one is shaken to the core. A sense of unravelling, disconnection and fear arise and may last for a short time, occur unexpectedly, or become a prolonged sense of "being unhinged". Patients, family members and health providers, experience groundlessness, albeit in different degrees. The experience of groundlessness leads to uncertainty and the quest for firm footing. This process is conceptualized as *Longing for Ground in a Ground(less) World*, a wish to minimize the uncomfortable or anxiety-provoking instability of groundlessness. Longing for ground is enacted in three overlapping ways: by turning toward one's discomfort and learning to let go (*engaging groundlessness*), turning away from the discomfort, attempting to keep it out of consciousness by clinging to familiar thoughts and ideas (*taking refuge in the habitual*), and learning to live within the flux of instability and unknowing (*living in-between*).

Findings from this study contribute to understanding how the processes of existential suffering are experienced and managed by patients, families, and health care providers. The core process of *Longing for Ground in a Ground(less) World *is congruent with Irvin Yalom's [23] theorizing of existential struggle. Yalom's work on existential concerns [23] and facing the terror of death [24] is rooted in his work as a psychiatrist and psychoanalyst. His premise, like many other scholars', is that fear of death is a primordial source of anxiety. Yalom [23] asserts that there is a basic human conflict "that flows from the individual's confrontation with the givens of existence" (p. 8). These givens are ultimate concerns that arise when a person is faced with mortality through illness, profound loss, or from deep reflection on what it means to be human.

According to Yalom [23], these ultimate concerns include: a) the tension between the inevitability of death and the wish to continue to be, b) the terrifying realization that "beneath us there is no ground" (p. 9) and therefore we are primarily responsible for, indeed are the authors of, our own world, choices and actions, c) the harsh reality that we are born alone and must die alone, and d) the realization that if death is inevitable and we have the freedom to constitute our world and are ultimately alone, then what is the purpose of life? What meaning does life have? When people come face-to-face with such concerns, it "permits raw death anxiety to erupt into consciousness" (p. 44), an experience that one participant described as "being shaken to the core". Supporting Yalom's [23] theorizing, our findings highlight the existential tension between the "confrontation with groundlessness and our wish for ground and structure" (p. 9).

It is important for clinicians to consider that if existential concerns are inherent in being human, then all patients may address them to some degree, in their own fashion, either consciously or unconsciously when faced with a serious illness. As Yalom [23] suggests, each person experiences the demands of confronting these concerns and the groundlessness that ensues, and this happens in highly individualized ways. A qualitative study by De Faye et al. [25] reports patterns of coping with stressors including existential distress for terminally ill individuals with cancer that align with the findings of our study. In particular, De Faye et al. identified emotion-focused approaches (e.g. catharsis), emotion-focused avoidance (e.g. distancing), and problem-focused approaches (e.g. direct action). Although Yalom suggests a universal, albeit individual, nature of the experience of groundlessness at the end of life, this does not imply that existential concerns will be paramount, conscious, or even open for discussion by all patients or health care providers. Nevertheless, by accepting an assumption that existential "facts of life", as Yalom describes them, are part of the terrain of sickness and death, health providers can attune themselves to patients who do wish to engage these concerns obliquely or straight on. Health care providers can also become aware of their selective inattention in the face of their own existential tensions or when with patients.

Although Yalom [23] describes groundlessness as reflecting a sense of meaninglessness, our findings take a broader view. Engaging groundless is a way of facing and leaning into the experience of loss, confusion, fear and uncertainty where loss of meaning is implicated. The compelling quest to make sense and reconstruct one's sense of self and life when it has been unravelled can be understood as a basic striving to find purpose and meaning. As a way of engaging groundlessness, notions of re-hinging one's life through meaning making and re-generating purpose in life are frequently associated with existential suffering [4,5,26,27].

Previous research into existential issues of patients with serious illnesses emphasizes the importance of meaning-making and redefining one's purpose in life [14,28,29]. In a grounded theory study, Sarenmalm and colleagues [30] explored the main concerns of twenty women with recurrent breast cancer. They described the process of making sense of living under the shadow of death as the core category illustrating the importance of meaning-making and finding new purpose as conditions change. The women's capacities to live in the present, not dwelling on the past or future, allowed them to find new ways of being, growing, and creating wellness. Our theorizing supports this finding and highlights the need for patient willingness and readiness to engage in these ways.

Whereas the emphasis in meaning making is on creating new understandings and identities, *taking refuge in the habitual *is a related yet contrasting process of holding on and retreating from engaging directly. This finding is supported by Yalom's [23] view that although humans experience death anxiety, a constant awareness would render us unable to function in the every-day. He suggests that fear "must be properly repressed to keep us living with any modicum of comfort" (p. 189) and that most people develop their own ways of discerning how much they can handle. The wish to hold onto what is known, including one's sense of self-identity, even when old patterns and ideas no longer work, is a process that can be both useful and constraining. Holding on to what is familiar, and the wish to return to what was normal, is an important issue described in the literature [31]. Consequently, this points to the need for health providers to be attentive to how patients and families narrate their experiences, how much they want to hear, and how "the way things are" may change (or not) as conditions change.

Whereas leaning into fear and anxiety and turning away towards familiar patterns are presented as distinct ways of managing suffering, living in-between is a paradoxical and recursive process that more closely contains opposites. This finding emphasizes a fluid and dynamic understanding of people's experience. Although empirical evidence suggests that positive personal changes may follow a confrontation with death, this experience may be transient and ungraspable. As one participant shared, "my life has changed profoundly and yet it's still the same". Others shared how they felt relief when their spouses once again recovered from a medical crisis, *and yet *they also confessed feeling frustrated, wishing to "get on with [their] life" after years of uncertainty. This complex experience of being both relieved-and-disappointed speaks to the complexity of experiences such as feeling both peace-and-anxiety, or being grounded-and-groundless. The ambiguous or liminal quality of serious illness is reported elsewhere [32,33].

This has implications for health providers who may gravitate towards the transformational possibilities of encounters with mortality without leaving room for less optimistic possibilities of resistance, anger, and despondency that may concurrently arise. Yalom [23] describes how "death is the condition that makes it possible for us to live life in an authentic fashion" (p. 31). However, even as the transformative possibilities of existential distress are reported in the literature [30], Yalom cautions not to be naive about how fraught with fear and anxiety the realization of mortality is. Living in-between compels health providers to acknowledge the complexity of fear and anxiety while allowing space for the uniquely dynamic nature of these processes for each person.

## Conclusion

Findings suggest that existential concerns are inherent in being human. Resultant theorizing emphasizes a fluid and dynamic understanding of existential suffering and compels health providers to acknowledge the complexity of fear and anxiety and the uniquely dynamic nature of these processes for each person. According to grounded theory methodology, theorizing is ongoing and open to continual revision. Further exploration with people in the midst of existential suffering is needed to expand current core concepts. While this goal poses ethical considerations and pragmatic challenges, further research into the nature of groundlessness and longing for ground would assist in refining the conditions and characteristics that lead to the three processes identified here. In addition, further understanding of how health care institutions can support health professionals to recognize and selectively attend to their own discomfort and abilities in order to assess and skilfully enter into conversations with patients and families is warranted.

## Competing interests

The authors declare that they have no competing interests.

## Authors' contributions

AB, RS, and PB designed the study and conducted the interviews and analysis; OP participated in analysis and manuscript preparation; all authors read and approved the final manuscript.

## Pre-publication history

The pre-publication history for this paper can be accessed here:

http://www.biomedcentral.com/1472-6955/10/2/prepub
